# Hospital Meetings, &c.

**Published:** 1900-03-03

**Authors:** 


### HOSPITAL MEETINGS, &C.

### THE METROPOLITAN HOSPITAL.

#### Festival Dinner: The Record Beaten.

The festival dinner of the Metropolitan Hospital, Kingsland Road, N.E., was held at the Hotel Metropole on Thursday evening, February 22nd, the Lord Mayor (Alderman A. J. Newton) presiding, in the unavoidable absence of both Mr. Alderman and Sheriff Bevan and Mr. Alderman and Sheriff Treloar. There was a large company. On his Lordship's right sat Lord Battersea, the treasurer, and on his left Mr. C. J. Thomas, the chairman of the hospital, among many others present being Sir J. Fortescue-Flannery, M.P., Mr. E. Flower, M.P., and Mr. Under-Sheriff Langton.

The usual loyal toast having been honoured, Mr. J. R. Pike gave "The Lord Mayor and the Corporation of the City of London," remarking that although situated outside the City the Metropolitan was, in its inception, essence, and support, truly a City hospital.

The Chairman, responding in his official capacity, said there was no city in the world that was better kept or better guarded, but during the last few weeks it had risen to Imperial heights and had deserved of its citizens their warmest thanks. (Cheers.)

In giving "Prosperity to the Hospital," the Lord Mayor said that at the present time the voluntary subscriptions in connection with the Transvaal War Fund, the Refugee Fund, the Volunteer Fund, and the Indian Famine Fund amounted to no less than nearly a million and a quarter of money. They might not unreasonably suppose that minor claims might tend to be overlooked. There was undoubtedly a fear that objects such as this hospital might be overlooked. He was satisfied, however, that when the claims of this hospital came to be considered their recollections of duty must prevail, and they must remember that the charitable institutions in their midst must be considered and attended to. The Metropolitan was essentially a City charity. Every Lord Mayor in turn was president of the hospital. Railway extensions had necessitated its removal, and at the present time the institution was situated in the centre of a densely-populated and very poor neighbourhood, including Shoreditch, Hoxton, Bethnal Green, Dalston, and Stoke Newington. It was built to accommodate 160 beds, but owing to lack of funds the committee had never been able to open more than 78 of those. During last year 867 in-patients were treated, and the attendances of out-patients reached the enormous total of 101,597. When they reflected for a moment what those figures meant they had occasion to very seriously consider the enormous benefits which the institution had conferred, and they must be satisfied that the interests of the institution deserved to be considered and to meet with a hearty response. Facts of such eloquence should appeal to those present for that support which he was satisfied the hospital merited, and which, he was sure, would be forthcoming. (Cheers.)

The toast was enthusiastically honoured.

Lord Battersea, whose name was associated with the toast, thanked the Lord Mayor for the sympathetic, enthusiastic, and gracious manner in which he had taken the place which was to have been filled by others, and remarked that his Lordship had fulfilled the most splendid traditions of the Mansion House. Proceeding to refer to the claims of the hospital, the speaker said that in the district in which it was situated there were 250,000 men, women, and children, nearly all of the working classes, and many of these families had sent men to fight for their country in South Africa. There was no other general hospital within two miles to which these poor people could go in times of sickness. As the Lord Mayor had said, the hospital was built to accommodate 160 beds, but, owing to the poorness of their resources, only 72 of these could at present be occupied. On that splendid freehold property in the Kingsland Road they had room for not 160, but 300, and if the Lord Mayors of the future could catch from their chairman of that evening some of his enthusiasm and some of his courage, he had little doubt that in a few years those 300 beds would be provided and filled. It was only necessary for the subject to be well ventilated, and the hospital to be well known, for the generosity of the City and of London to help them in the splendid manner in which they were helping other institutions. In the great area indicated they had only the possibility of supplying one bed to something like 3,000 of the population, whereas, the needs of the case required one bed to every 300 of the population. He therefore commended the hospital to their good-will. He knew of no hospital in London that was more deserving of their regard and support, and, while thanking the patrons of the institution for the generous manner in which they had supported it in the past, felt every confidence in pleading for their continued support. If they would only study the interests of the case and watch the work done at the hospital, they would find that work well done, that the medical staff was able, brilliant, and competent, and that their nurses were amongst the most excellent in the land, which was saying a good deal. (Cheers.) The work done in the hospital was excellent, the cause was a splendid one, economically carried on, and if those whom he was addressing would assist it, and induce their friends to do the same, he was sure they would never have cause to regret it. (Cheers.)

Sir Fortescue Flannery, M.P., in proposing "The Committee of Management," referred with great gratification to the harmony which prevailed on that body, and to the good work done not only by the hospital itself, but by the convalescent home in connexion with it at Cranbrooke, Kent.

Mr. C. J. Thomas (chairman of the hospital), in reply, said the condition of the institution from a medical, surgical, and nursing aspect was all that could possibly be desired. Ever since it was established it had been favoured with very great talent in these departments. The balance-sheet showed an income last year of nearly £10,000, which was rather more than the expenditure. They had still, however, a debt with which to struggle, and, although it had been reduced in the past year by between £300 and £400, the reduction was not so great as in the previous year. They would all like that debt cleared off. In addition to that income they also had secured to them early in the year, through the interest of their good friend Mr. Scales, the important sum of £1,000, which had been invested. Mr. F. S. Franklin, a new member of the committee, had been instrumental in raising no less than £1,660. The Hospital Sunday Fund Committee had increased their contribution from £487 to £729, and the Hospital Saturday Fund Committee had raised theirs from £134 to £146. They attached value to these increases, because they were made only after careful examination and inquiry, and because the hospital had been found worthy of such substantial increases.

The Secretary then announced the list of subscriptions and donations in connection with the festival, mentioning *inter alia* that a gentleman who wished to remain anonymous offered £100 at the beginning of the year if nine other gentlemen could be found to give the same by the end of the present month, and this £1,000 had been raised. (Cheers.) The best previous total, when Mr. Leopold Rothschild was in the chair, was £3,341, but this year the total reached £4,358. (Loud cheers.)

"The Hospital Staff," "The Chairman," and "The Visitors" were the other toasts, and there was an enjoyable musical programme.

### CENTRAL LONDON OPHTHALMIC HOSPITAL.

The fifty-sixth annual general meeting of the Central London Ophthalmic Hospital was held on Thursday, February 22nd, Mr. J. S. Burrows presiding. The report presented showed ordinary income of £1,636, and ordinary expenditure £1,590, plus £32 for repairs. During the year 11,925 new cases were admitted—a considerable increase on the preceding twelve months. The in-patients numbered 402 —92 more than in 1898. The attendances numbered no less than 30,275. To meet the rapidly-increasing demands on their space the committee had bought two of the adjoining houses and adapted them for use; but the buildings were old, and there was no doubt the hospital ought to be entirely reconstructed. That this view is shared by the Prince of Wales's Fund is shown by a special grant towards the building fund. Other contributions to this fund brought the total during the year to £464, so that the balance to credit now stood at £2,730. The plans for rebuilding could not, however, be commenced until at least £5,000 had been obtained, as without this a renewal of the leases would not be granted.

The Chairman moved the adoption of the report and accounts, and laid stress on the fact that the increase in the work during the year had, of course, entailed added expense, and made the need for rebuilding more urgent. He considered that the hospital was one of the best of its kind in London, and deserving of all support. The motion was seconded by Colonel Tatham, and agreed to, as was also a proposal of a hearty vote of thanks to the surgical staff.

### CITY OF LONDON LYING-IN HOSPITAL.

The annual court of governors of the City of London Lying-in Hospital, City Road, was held at the hospital on February 21st, at three o'clock. The Rev. J. H. Parry, rector of St. Luke's, took the chair, and moved the adoption of the report.

The report, which had previously been circulated, was taken as read, and carried unanimously. It stated that the year had been a satisfactory one in every respect. The largest number of patients since 1840—namely, 585—had been admitted to the wards, amongst whom only one death had taken place. This, too, occurred from phthisis, eighteen days after the confinement, with which it was in no way connected. Of the 593 children born 315 were boys and 278

girls; 31 infants were stillborn, and 17 died. The average number of beds occupied were 25·5, and the length of each patient's stay was about sixteen days. Owing to far-reaching changes in the immediate neighbourhood, which is gradually replacing a residential population by a commercial one, fewer out-patients have been attended during the past twelve months than in the previous year. It is therefore proposed to add South Tottenham to the list of districts coming within the ministrations of the charity. The number of cases attended in their own homes was 1,559, that of the children born 1,582, of whom 780 were boys and 802 girls. Six women and 29 infants died, and 70 babies were stillborn. The school for midwives and monthly nurses has shown satisfactory results—38 of the 48 midwifery pupils presented themselves for examination, and obtained the L.O.S. certificate, and 159 monthly nurses received training. The new building for the accommodation of the pupil nurses is complete, and will be occupied as soon as it is furnished. It contains 23 cubicles, a comfortable sitting-room, and it is fitted with baths, lavatories, and other modern conveniences. It is well ventilated, and is heated throughout by hot-water radiators. Mr. H. H. Collins was the architect, and Messrs. Falkner executed the work for £2,952. The labour ward has been refloored with pitch pine at a cost of £32, and inlaid linoleum has been put down in two wards as an experiment, the outlay being £12 each. The income for the year was £4,220, the ordinary expenditure £3,100, the extraordinary £2,608. £2,000 Consols were sold to meet the expenses of the new buildings, and there is a deficit in the year's total accounts of £1,489. At the request of the Princess Louise, president of the District Branch of the Soldiers and Sailors' Families Association, wives of men with the Colours in South Africa will receive the benefits of the hospital without subscribers' letters. Dr. F. Godfrey has been elected hon. life governor of the hospital upon resigning his appointment as one of the district surgeons in recognition of his long services. His successor is Dr. C. Beauchamp Hall. A vote of condolence was sent to the son of the late Mr. Charles Gordon, vice-president of the charity. Mr. Gordon was an active and valuable friend to the hospital, and took the keenest interest in every detail of work, structure, and management. The committee, hon. officers, and surgeon-accoucheur were re-elected, and the proceedings terminated.

### KING'S COLLEGE HOSPITAL.

The sixty-first annual meeting of King's College Hospital was held on Thursday, 22nd inst., Viscount Dillon presiding. The report presented by the committee of management stated that the past year had been one of progress and prosperity. The whole of the wards had been re-floored with polished teak, and electric light supplied to those not already so lit. These improvements had involved a capital expenditure of £3,065, causing a deficit in the year's accounts of £1,464. The carrying out of the work had necessitated the closing of the hospital for nine weeks. This was done during the summer vacation, when the work was always lighter; and arrangements were made with other hospitals and convalescent homes to receive those patients not well enough to return home. Notwithstanding this interruption the in-patients numbered 2,408, as compared with an average of 2,500, so that the benefits of the hospital were not seriously diminished. In the out-patient department the new cases numbered 16,718, of which 7,687 were accidents; 412 poor married women were attended at their own homes during confinement. There was a daily average of 168 beds occupied. The ordinary income for the year was £14,049; the legacies £4,567. The ordinary expenditure amounted to £16,504; the extraordinary expenditure (floors, electric lighting, chapel, and convalescent home) to £3,576. The total receipts (£18,616) were thus £1,112 in excess, and the total disbursements (£20,081) £1,622 more than in 1898. The committee expressed their gratitude at the substantial help afforded by a grant of £1,175 (£175 being a special donation towards the contemplated provision of new out-patient and casualty departments) from the Prince of Wales's Hospital Fund; of £1,666 from the Sunday Fund; and of £329 from the Saturday Fund. The festival dinner in May last produced £2,150. The Hon. W. F. D. Smith, M.P., had consented to p[illegible]le at the forthcoming dinner on May 7th, when a special effort would be made in view of the need for extinguishing the debt on the reflooring account. The following changes had taken place in the medical staff:—Dr. Burney Yeo, who had rendered valuable services to the hospital for many years, had been appointed by the council of King's College consulting physician to the hospital; Sir Hugh Beevor, M.D., had been appointed physician to the hospital, Dr. W. Aldren Turner assistant physician, Dr. G. Frederic Still assistant physician for diseases of children, and Mr. C. E. Wallis assistant dental surgeon. From the surgical staff of the hospital Professor W. Watson Cheyne, Mr. George L. Cheatle, and Mr. L. Vernon Cargill had been selected for service at the seat of war; from the nursing staff one of the ward sisters and a lady formerly one of their ward sisters had also gone out. The committee had consented to place fifteen beds at the disposal of the War Office for sick and wounded soldiers.

The proceedings were brief, consisting of the adoption of the report and accounts, the reappointment of the treasurer and committee, and the passing of cordial votes of thanks for their past services; with similar compliments to the auditors and medical and nursing staffs, and to the chairman for presiding.

### CHARING CROSS HOSPITAL.

In the absence of Mr. Beerbohm Tree, Mr. Edward Terry presided on Wednesday, 21st inst., at the seventy-ninth annual general Court of Governors of the Charing Cross Hospital. He said that when, in the kindness of his heart, Mr. Tree promised to take that chair, he had no idea, especially in the weather they had been enjoying, he would be appearing in "A Midsummer Night's Dream"; but so it was, and that being so, he had been appointed his understudy. He filled his post with a great sense of diffidence and unworthiness, and hoped that with the council's report before them the Governors would be able to imagine that what he (Mr. Terry) did not say Mr. Tree, had he been there, would have said. So, reading between the lines, they would be able to make up a really capital speech. He thought at any rate that they might congratulate themselves on having had a very glorious year—1898 had been considered a splendid year of progress, but last year had certainly not been behind it. The increases in their subscriptions and donations showed the really strong hold they had on the public. And in connection with the past year one name at least would always remain dear to the Governors of that institution—the name of Mrs. Arthur Paget, who, as secretary of the Ladies' Committee, had done so much, with those who had assisted her, to make the Albert Hall Bazaar the brilliant success it was. From his own acquaintance with the theatrical profession, he could form some idea of the work and energy needed to achieve so magnificent a sum as £16,000, and on behalf of the hospital and of those who benefited by it he tendered their heartfelt thanks. Last year, at that meeting, the Bishop of London referred to the absolute need for increased accommodation. Thanks to their friends they had, during the past year, progressed admirably in that direction, and hoped this year to go still farther forward. As to their current work, they had during 1899 cared for nearly 25,000

patients at a cost of between £14,000 and £15,000, and when they got the increased accommodation they would be able to do more and to do it better. Many of their friends were greatly exercised lest in this year of Funds their institution might suffer; but when he remembered how often the wives and families of our brave sailors and soldiers, as well as they themselves, had been helped in their sorest need by the Charing Cross Hospital, he felt that its claims would not go unheeded.

The report of the council stated that the prosperity of the past year was in a great measure due to the wonderful success of the grand bazaar at the Albert Hall on June 21st and 22nd, in aid of the Special Appeal Fund. The total amount raised by this effort was £16,020, and very full details regarding it were included in the report. The receipts for the year were: For the general fund (including legacies), £18,067; for the special appeal, £19,153; for the convalescent home, £877. Annual subscriptions showed an increase from £2,295 to £2,360. The expenditure on maintenance and administration totalled £14,489; analysis of this expenditure after deducting £2,252, estimated cost of 22,527 out-patients, gave £69 18s. as the cost per bed, and £5 5s. as the cost per in-patient. The number of patients treated during the year amounted to 24,853. There were 2,326 in-patients, 9,262 out-patients, and 13,265 cases of accident and emergency. This increase, as compared with the previous year, was due to the fact that King's College and Westminster hospitals were closed for some weeks during the summer. During the year 383 patients were received at the convalescent home, as against 309 in 1898. Of these 127 had not passed through the hospital. Thirty beds were available for invalid soldiers from South Africa should they be required. The handsome sum realised at the bazaar had enabled the council to at once proceed with the plans for the new buildings. Competitive designs had been submitted by four well-known architects. After careful consideration, during which the council received valuable assistance from Mr. William Wallace, F.R.I.B.A., Mr. A. Saxon Snell, F.R.I.B.A., was selected as the architect who had, on the whole, submitted the most suitable set of plans. These plans, however, required considerable modification. The configuration of the site available made this task very difficult, and a final revision of the plans was not arrived at until November. Meanwhile it was necessary that new boilers should be provided, as they were advised that those which had been in use for 18 years would probably not last out the winter. The completion of the new boilers necessitated the erection of a permanent chimney shaft as soon as possible; and, as a measure of economy, it was decided to build the new grand staircase at the same time, and, in addition, the necessary arrangements were being made for providing at once new sanitary conveniences for the existing wards, and a new kitchen. Mr. Snell had also been instructed to prepare the working plans for the new building on the King William Street side of the block, and the council hoped their next report would give an account of substantial progress with the long-wanted and urgently-needed improvements. During the year they had purchased from the Crown the freehold of Nos. 17 and 18, Chandos Street, so that the freehold of all the houses next the hospital in this street now belonged to them. In addition to the Albert Hall bazaar, thanks were due to the Ladies' Committee for organising a very successful fancy dress ball at the Empress Rooms, Kensington Palace Hotel, in aid of the Special Appeal Fund. The ball was held on December 11th, and produced a profit of £300. The council deeply regretted to have to report the death, after a very short illness, of Dr. Charles J. Arkle, the junior assistant physician to the hospital. Dr. Arkle was appointed in March, 1895, to the vacancy caused by Dr. Montagu Lubbock's resignation. He had been for the previous three years pathologist and curator of the museum. By his untimely death the hospital had lost a valuable officer and the school an excellent teacher. Dr. James Galloway, the physician for skin diseases, was appointed an assistant physician on the general staff of the hospital, and Dr. William Hunter was appointed physician in charge of the electrical department, to fill the vacancies caused by Dr. Arkle's death. Acting on the recommendation of the Medical Committee the council had created the appointment of bacteriologist to the hospital and lecturer on bacteriology to the medical school, with a salary of £100 a year. Dr. J. W. H. Eyre had been elected to the office. Dr. F. W. Richardson having resigned the post of resident medical officer, the vacancy was filled by the appointment of Mr. E. C. Montgomery, an old student of the hospital, who had held the offices of house physician and house surgeon. The council concluded their report with an expression of anxiety as to the prospects of the coming year. The demands on the public for help for the sufferers by the war have been so urgent, and so largely responded to, that they could not but feel apprehensive lest the claims of existing charities might suffer. Nevertheless the support they had received during the past few years encouraged them to hope that their efforts to improve the hospital and to put it into a satisfactory condition would continue to meet with the generous assistance of the public. For the maintenance of the hospital and of the convalescent home a sum of £16,000 is required every year, of which, during the present year, the council expected to receive from ordinary sources, including unpaid legacies, about £10,000, leaving £6,000 to be provided for from donations and legacies. For the structural improvement, the council began the year with £25,000 in hand or promised, but at least £50,000 more would be required to complete the improvements. The report and accounts having been adopted, votes of thanks were passed to the various committees for their services. The retiring members of the council and the auditors were re-elected, and a vote of thanks to the chairman brought the proceedings to a close.

### NEWSVENDORS' BENEVOLENT INSTITUTION.

The sixty-first annual meeting of the Newsvendors' Benevolent and Provident Institution was held at the Memorial Hall, Farringdon Street, on Tuesday, 20th inst. Lord Glenesk, one of the presidents, was announced to preside, but did not arrive till late in the proceedings, being detained in the House of Lords. In his absence Sir John Hutton took the chair. The report presented stated that the past year had been one of the most prosperous in the annals of the institution: £1,300 was contributed at the festival last year, presided over by Lord Rosebery; and in addition the committee had received from Mr. H. B. Marshall the capital of "The Horace Marshall Pension Fund"; and also an annual grant from the proprietors of The Hospital in creation of The Hospital pensions. Altogether, the net accession to the funds of the institution during the year amounted to £2,328.

Sir John Hutton, in moving the adoption of the report, said they were now in the happy position of benefiting thirty-six pensioners, all respectable people who, through stress of circumstances, had been obliged to come to them for assistance.

Three pensioners were then elected, and a resolution was adopted increasing pensions payable out of certain funds by £5 each. The following additional rule was also adopted and ordered to be forwarded to the Registrar of Friendly Societies for registration:—

"Rule 8 (c)—'The Hospital' Pensions.—Sir Henry Charles Burdett, K.C.B., on behalf of The Hospital, having promised to annually contribute £35 for the purpose of granting £20 to a man and £15 to a woman, such grants to be called 'The Hospital' Pensions, the committee shall in each and every year, immediately after the receipt of such contribution, elect such candidates from amongst the eligible benefit members of this institution of five years standing or upwards, and 55 years of age or upwards, or the widows of like age of such members, such recipients to hold such grants for one year and receive the same by four quarterly payments; the committee to notify such elections to Sir Henry Burdett, or his nominee, and also to the then next general meeting of the institution."

The Chairman said the committee had selected Charles Skeats to be the recipient of a £20 "The Hospital" Pension, and Elizabeth Gander to be the recipient of a £15 "The Hospital" Pension for the current year, and the secretary (Mr. Wilkie Jones) was instructed to forward to Sir Henry Burdett a notification of these elections, with an expression of thanks for the generous assistance thus rendered to the objects of the institution.

Various officers were re-elected, and votes of thanks closed the proceedings.

